# Cost and Access Changes Appreciated with TNFa Inhibitor Biosimilars

**DOI:** 10.1093/geroni/igaf122.3741

**Published:** 2025-12-31

**Authors:** Joseph Visingardi, Bryan Sherwood

**Affiliations:** Albany Medical College, Albany, New York, United States; Dartmouth Hitchcock Medical Center, Lebanon, New Hampshire, United States

## Abstract

Biologic therapies, such as adalimumab, rank among the most costly drugs in the United States. This study aimed to project how pricing and access to adalimumab may shift once it loses patent protection, using the experiences of other biologics that have already gone off-patent as a reference. To evaluate market dynamics, CMS Medicare expenditure data from 2017 through 2021 were examined for several branded biologics and their biosimilars. Analyses focused on changes in unit cost, the number of beneficiaries using the branded product, and shifts in the total population using either branded or biosimilar versions. During this period, branded adalimumab—without biosimilar competition—saw a 72% increase in cost per unit alongside a 23% rise in beneficiary numbers. In contrast, branded infliximab, which faced biosimilar competition, experienced a 53% decline in unit cost, a 26% reduction in branded drug users, and a modest 1% overall decline in total beneficiaries across all formulations. Similar patterns were observed with most other drugs analyzed, showing decreased costs and reduced reliance on branded versions, but with mixed results in overall patient uptake across both branded and biosimilar products. These findings suggest that the loss of exclusivity for biologics, including adalimumab, generally results in lower prices and diminished market share for the brand. However, whether these shifts translate into improved affordability and access remains uncertain, as the anticipated increase in total beneficiaries following the introduction of lower-cost biosimilars was not consistently observed.

